# Synergistic apoptotic and epithelial–mesenchymal transition-inhibitory effects of *Hericium erinaceus* extract in canine mammary cancer cell lines

**DOI:** 10.14202/vetworld.2026.2318-2338

**Published:** 2026-06-05

**Authors:** Usuma Jermnak, Aksorn Saengtienchai, Tassanee Jaroensong, Sunee Kunakornsawat, Sirikul Soontararak, Wachiraphan Supsavhad, Kannika Siripattarapravat, Anurak Khieokhajonkhet, Yared Beyene Yohannes

**Affiliations:** 1 Department of Pharmacology, Faculty of Veterinary Medicine, Kasetsart University, Bangkok, 10900, Thailand; 2 Department of Companion Animal Clinical Sciences, Faculty of Veterinary Medicine, Kasetsart University, Bangkok, 10900, Thailand; 3 Department of Pathology, Faculty of Veterinary Medicine, Kasetsart University, Bangkok, 10900, Thailand; 4 Center for Agriculture Biotechnology, Faculty of Agriculture, Natural Resources and Environment, Naresuan University, Phitsanulok, 65000, Thailand; 5 Department of Veterinary Forensics, Faculty of Veterinary Medicine, Hokkaido University, Sapporo, 060-0818, Japan; 6 Laboratory of Toxicology, Department of Environmental Veterinary Science, Faculty of Veterinary Medicine, Hokkaido University, Sapporo, 060-0818, Japan

**Keywords:** apoptosis, canine mammary cancer, epithelial–mesenchymal transition, *Hericium erinaceus*, medicinal mushroom, metastasis inhibition, phytochemicals, selective cytotoxicity

## Abstract

**Background and Aim:**

Canine mammary cancer (CMC) is one of the most common malignant neoplasms in female dogs, with high metastatic potential and limited therapeutic options. Natural bioactive compounds derived from medicinal mushrooms have gained increasing attention because of their anticancer properties and low toxicity. *Hericium erinaceus* (HE) is a medicinal mushroom known for its antioxidant and antitumor activities; however, its anticancer effects in CMC remain poorly understood. Therefore, this study investigated the *in vitro* antiproliferative, pro-apoptotic, and epithelial–mesenchymal transition (EMT)-inhibitory effects of HE methanolic extract in two CMC cell lines, CHMp-13a and CHMp-5b.

**Materials and Methods:**

The anticancer activity of HE methanolic extract was evaluated using CHMp-13a and CHMp-5b CMC cell lines, while Madin-Darby canine kidney cells were used as normal controls. Cell viability was assessed using the 3-(4,5-dimethyl-2-thiazolyl)-2,5-diphenyl-2H-tetrazolium bromide assay. Cell migration and invasion were evaluated using wound healing and Transwell assays, respectively. Apoptosis was analyzed using Annexin V-fluorescein isothiocyanate/propidium iodide flow cytometry. Relative mRNA and protein expression levels of apoptosis- and EMT-related markers were determined using quantitative real-time polymerase chain reaction and western blotting. The phytochemical profile of the extract was characterized using liquid chromatography quadrupole time-of-flight mass spectrometry.

**Results:**

HE extract significantly inhibited proliferation of both CMC cell lines in a dose- and time-dependent manner, with greater selectivity toward CHMp-13a cells and minimal cytotoxicity in normal cells. Morphological analysis revealed apoptotic features, including cell shrinkage, detachment, and cytoplasmic vacuolization. The extract significantly suppressed migration and invasion capacities of both CMC cell lines. Flow cytometric analysis demonstrated increased apoptotic cell populations following treatment. Molecular analyses showed upregulation of the pro-apoptotic marker BAX and downregulation of the anti-apoptotic marker BCL-2. Furthermore, HE extract suppressed EMT progression by increasing E-cadherin expression while reducing N-cadherin expression. Phytochemical screening identified 17 bioactive compounds, including erinacines, hericenones, hericene derivatives, and phenolic compounds, which may contribute to the observed anticancer activities.

**Conclusion:**

HE extract demonstrated potent *in vitro* anticancer activity against CMC cells through synergistic induction of apoptosis and suppression of EMT-associated metastatic behavior. These findings suggest that HE extract may serve as a promising natural adjuvant candidate for the management of CMC and warrants further *in vivo* and mechanistic investigations.

## INTRODUCTION

The prevalence of age-related diseases in companion animals has increased markedly over the past decade. This trend is largely attributed to improved pet longevity, which consequently increases susceptibility to chronic disorders such as cardiovascular disease, neurodegeneration, and various forms of neoplasia [1, 2]. Among these disorders, neoplastic diseases remain the leading cause of mortality in senior companion animals [3]. In female dogs, mammary gland tumors are the most frequently diagnosed neoplasms worldwide, with approximately 50% of these tumors reported to be malignant [4]. A notably high prevalence of canine mammary tumors has also been documented in Thailand, accounting for 24.5% of all canine biopsy specimens, of which 85.5% were classified as malignant [5]. Canine mammary cancer (CMC) shares several clinical, histopathological, and molecular characteristics with human breast cancer, making it an important comparative model for translational oncology studies [6]. Several factors contribute to the development and progression of CMC, including age, hormonal exposure, genetic predisposition, environmental influences, and nutritional factors [7]. The aggressiveness and clinical outcome of mammary tumors are strongly associated with cellular pleomorphism, mitotic activity, lymphatic invasion, and metastatic dissemination to regional lymph nodes [8].

Cancer progression is characterized by the acquisition of multiple mechanisms that enable tumor cells to survive, proliferate, invade surrounding tissues, and metastasize to distant organs [9]. One of the fundamental hallmarks of cancer involves the evasion of apoptosis, a highly regulated process responsible for maintaining tissue homeostasis through the elimination of damaged or potentially oncogenic cells [10]. Dysregulation of apoptosis-related genes may prevent programmed cell death, thereby facilitating uncontrolled cancer cell proliferation and tumor progression [11]. In particular, decreased expression of the proapoptotic protein BAX together with increased expression of the antiapoptotic protein BCL-2 suppresses apoptotic signaling pathways and promotes cancer cell survival [12]. Another critical mechanism associated with tumor progression is EMT, which enables epithelial cancer cells to acquire migratory and invasive properties [13]. During EMT, epithelial markers such as E-cadherin are downregulated, whereas mesenchymal markers including N-cadherin and SLUG are upregulated, resulting in enhanced motility, invasiveness, and metastatic potential [14, 15]. In CMC, activation of EMT is strongly associated with aggressive tumor behavior and poor prognosis.

Early diagnosis and effective therapeutic intervention are essential for improving survival outcomes and quality of life in dogs affected by mammary cancer. Currently, surgical excision remains the primary treatment strategy for localized mammary tumors, whereas chemotherapeutic agents such as doxorubicin (DOX) and 5-fluorouracil are frequently used in advanced or metastatic cases [16-18]. Nevertheless, conventional chemotherapeutic regimens often exhibit limited efficacy and may induce severe adverse effects, including cardiotoxicity, gastrointestinal disturbances, nephrotoxicity, and myelosuppression in canine patients [19]. Therefore, the development of safer and more effective therapeutic or adjuvant agents capable of suppressing tumor growth and metastasis while minimizing toxicity remains a major challenge in veterinary oncology.

Natural products derived from medicinal mushrooms have attracted considerable scientific attention because of their diverse pharmacological properties, including antioxidant, anti-inflammatory, immunomo-dulatory, antimicrobial, and anticancer activities [20, 21]. Several studies have demonstrated that mushroom-derived bioactive compounds exert antitumor effects through both direct cytotoxic mechanisms and modulation of immune responses [22]. In Asian countries such as Japan and China, medicinal mushrooms are approved and widely used as adjunctive therapies in cancer management, either in combination with chemotherapy and radiotherapy or as supportive single-agent treatments in humans [23]. Consequently, medicinal mushrooms have emerged as promising sources of novel bioactive compounds for pharmaceutical and anticancer drug development [24, 25].

*Hericium erinaceus* (HE) is an edible and medicinal mushroom commonly distributed throughout Asian countries and has been traditionally used as a safe herbal remedy with minimal toxicity [26, 27]. Previous studies have reported that HE extract possesses multiple biological activities, including antioxidant, neuroprotective, anti-inflammatory, and anticancer effects [28, 29]. In several human cancer models, HE extract has been shown to inhibit cell proliferation, suppress invasion and metastasis, induce apoptosis, and arrest the cell cycle. For example, the tumoricidal activity of HE extract was demonstrated in MCF-7 breast cancer cells in a time- and dose-dependent manner through apoptosis induction and G1 phase cell-cycle arrest [30]. In addition, several phytochemicals identified in HE, including erinacines, hericenones, and other terpenoid compounds, have been associated with anticancer and antioxidant properties. These compounds are believed to regulate multiple molecular pathways involved in tumor progression and metastasis.

Despite increasing evidence regarding the anticancer properties of HE extract in human cancer models, limited information is available concerning its therapeutic potential in canine cancers, particularly CMC. Most previous studies have focused predominantly on human-derived cancer cell lines, while the biological responses of CMC cells to HE extract remain poorly characterized. In particular, there is insufficient information regarding the effects of HE extract on distinct CMC phenotypes with varying invasive characteristics. Moreover, the molecular mechanisms underlying the anticancer activity of HE extract in CMC have not been comprehensively elucidated. Specifically, there is a lack of evidence regarding its role in regulating apoptosis-associated proteins such as BAX and BCL-2 and EMT-associated markers including E-cadherin, N-cadherin, and SLUG in CMC cells. Furthermore, the phytochemical composition potentially responsible for these biological activities in CMC models remains inadequately characterized. Although several bioactive compounds have previously been identified in HE, their contribution to apoptosis induction, EMT suppression, and antimetastatic activity in CMC cells has not yet been fully investigated. Therefore, additional studies are required to clarify the molecular pathways and phytochemical constituents associated with the anticancer effects of HE extract in CMC models.

Therefore, this study aimed to investigate the *in vitro* anticancer activity of HE extract against two distinct CMC cell lines, CHMp-13a and CHMp-5b, to provide a more comprehensive understanding of its therapeutic potential. Specifically, this study evaluated the effects of HE extract on cancer cell proliferation, morphological alterations, migratory capacity, apoptosis induction, and EMT-associated molecular changes. In addition, the expression patterns of apoptosis-related markers, including BAX and BCL-2, together with EMT-related markers such as E-cadherin, N-cadherin, and SLUG, were investigated at both mRNA and protein levels. Furthermore, the phytochemical profile of the HE extract was characterized using liquid chromatography quadrupole time-of-flight mass spectrometry (LC/Q-TOF/MS) analysis to identify potential bioactive compounds that may contribute to its observed anticancer activities in CMC cell lines.

## MATERIALS AND METHODS

### Ethical approval

This study was conducted entirely under *in vitro* conditions using the established CMC cell lines CHMp-13a and CHMp-5b, together with the Madin-Darby canine kidney (MDCK) cell line. No live animals, animal tissues, or clinical animal samples were used during any stage of the experimental procedures. Therefore, ethical approval from the Institutional Animal Care and Use Committee was not required. All experimental procedures were performed in accordance with standard biosafety and laboratory guidelines for cell culture-based research at the Faculty of Veterinary Medicine, Kasetsart University, Bangkok, Thailand.

### Study period and location

This study was conducted from December 2024 to December 2025 at the Kasetsart University Animal Cell Bank, Faculty of Veterinary Medicine, Kasetsart University, Bangkok, Thailand. Additional phytochemical analyses were performed in collaboration with specialized analytical laboratories equipped for LC/Q-TOF/MS-based compound characterization.

### Study design

This study was designed as an *in vitro* experimental investigation to evaluate the anticancer effects of HE extract against CMC cell lines. The experimental workflow included extraction of mushroom bioactive compounds, assessment of cytotoxicity and selective antiproliferative activity, evaluation of cellular morphology, migration inhibition assays, apoptosis quantification, analysis of apoptosis- and EMT-related gene and protein expression, and phytochemical characterization using LC/Q-TOF/MS. The CHMp-13a and CHMp-5b cell lines were used as representative low-grade and high-grade invasive canine mammary adenocarcinoma models, respectively, whereas MDCK cells were employed as a noncancerous control cell line for selectivity analysis.

### Mushroom material and extraction

The fruiting bodies of HE were obtained from Organic Sentang Hed Farm Limited Company, Phitsanulok, Thailand. A voucher specimen (BBH No. 50496) was deposited at the Fungarium of BIOTEC Bangkok Herbarium, National Biobank of Thailand, Thailand. The HE fruiting bodies were dried at 60°C and ground into a fine powder. Briefly, 200 g of HE powder was extracted with 95% methanol (MeOH) at a ratio of 10 g/100 mL and stored at 4°C for 24 h. The mixture was subsequently sonicated for 30 min and centrifuged at 10,000 × *g* for 10 min. The supernatant was filtered through filter paper and concentrated to dryness under reduced pressure using a rotary evaporator (Buchi, Flawil, Switzerland) at 40°C, yielding 24.55 g of crude HE extract (12.28% yield). The extract was stored at −20°C until further use. For biological assays, the crude extract was freshly reconstituted in dimethyl sulfoxide (DMSO) and immediately diluted in culture medium to ensure that the final DMSO concentration did not exceed 0.1% (v/v), thereby minimizing solvent-induced cytotoxicity.

### Cell lines and cell culture

The CHMp-13a (low-grade invasive canine mammary adenocarcinoma) and CHMp-5b (high-grade invasive canine mammary adenocarcinoma) cell lines [31] were kindly provided by Professor Dr. Takayuki Nakagawa, Laboratory of Veterinary Surgery, Graduate School of Agricultural and Life Sciences, The University of Tokyo, Japan. The MDCK cell line was obtained from the American Type Culture Collection (ATCC), Manassas, VA, USA.

CMC cells were cultured in RPMI-1640 medium (Corning, New York, NY, USA) supplemented with L-glutamine, 10% fetal bovine serum (FBS; Invitrogen, Carlsbad, CA, USA), and 1% antibiotic-antimycotic solution (Gibco, New York, NY, USA). MDCK cells were maintained in Dulbecco’s Modified Eagle Medium (DMEM; Corning, New York, NY, USA) containing 4.5 g/L glucose and L-glutamine supplemented with 10% FBS and 1% antibiotic-antimycotic solution. All cultures were maintained in a humidified incubator containing 5% CO_2_ at 37°C. Upon reaching approximately 80% confluence, cells were harvested using 0.25% TrypLE (Gibco) for subculturing.

In this study, CHMp-13a and CHMp-5b cells were used between passages 28 and 41, whereas MDCK cells were used between passages 103 and 107. To ensure experimental integrity and exclude contamination-associated bias, all cell lines were screened for mycoplasma contamination using polymerase chain reaction (PCR)-based detection assays. The results confirmed that all cell lines used in this study were free from mycoplasma contamination (Supplementary Figure S1).

### Cell proliferation assay

Cell proliferation was evaluated using the 3-(4,5-dimethyl-2-thiazolyl)-2,5-diphenyl-2H-tetrazolium bromide (MTT) assay according to previously described protocols [32, 33]. Cells were seeded into 96-well plates at a density of 7 × 10³ cells/well and allowed to adhere for 24 h. Subsequently, cells were treated for 48 h with various concentrations of HE extract (0-1000 µg/mL), 0.1% DMSO as the negative control, or DOX at 2 µg/mL as the positive control.

Following treatment, 10 µL of MTT solution was added to each well, and the plates were incubated for an additional 4 h. Thereafter, 100 µL of solubilization buffer was added to each well, followed by overnight incubation at 37°C. The absorbance of the solubilized formazan product was measured at 570 nm using a microplate reader (BioTek, Winooski, VT, USA). All experiments were conducted in quadruplicate across three independent experimental sessions. Blank well absorbance values were subtracted from all readings to eliminate background interference.

Cell viability was expressed as a percentage relative to the negative control group, which was defined as 100% viability, according to the following formula:

Cell viability (%) = [(At − Ab)/(Ac − Ab)] × 100

Where, At = absorbance of treated cells; Ab = absorbance of blank wells; and Ac = absorbance of control cells.

The half-maximal inhibitory concentration (IC_50_) values were calculated from dose-response curves. In addition, the selectivity index (SI) was used to determine the selective cytotoxicity of HE extract toward cancer cells relative to normal cells using the following equation:

SI = IC_50_ value (normal cells)/IC_50_ value (cancer cells)

### Cell morphology analysis by phase-contrast microscopy

Based on preliminary proliferation assays and IC_50_ values, concentrations ranging from 0 to 600 µg/mL were selected for further morphological evaluation of CHMp-13a and CHMp-5b cells. Cells were seeded into 6-well plates at a density of 4 × 10^5^ cells/well and treated with HE extract (0-600 µg/mL). After 48 h of incubation, cellular morphological alterations including changes in cell shape, adherence, shrinkage, and apoptotic features were examined and documented using a phase-contrast microscope (Nikon, Tokyo, Japan) at 10× magnification.

### Cell migration assays

Cell migration was initially evaluated using a scratch-wound assay according to previously described methods [32, 33]. Briefly, CMC cells were seeded into 6-well plates at a density of 4 × 10^5^ cells/well and cultured until complete confluence was achieved. A linear wound was generated using a sterile 100 µL pipette tip, after which cells were treated with various concentrations of HE extract (0-600 µg/mL). Wound healing was monitored at 0, 12, and 24 h after scratching. Images were captured using a microscope equipped with NIS-Elements D imaging software version 5.2 (Nikon, Tokyo, Japan). The average wound area was determined using linear measurements obtained from three equidistant positions across each wound. Cell migration was quantified by comparing wound areas at 12 and 24 h relative to the initial wound area at 0 h. Experiments were repeated four times.

The percentage of wound area was calculated as follows:

% wound area = (Wt/W0) × 100

Where, Wt = wound area at time t and W0 = wound area at 0 h.

The migratory capacities of CMC cells were further evaluated using a 24-well Transwell insert system with an 8 µm pore membrane (Corning). CHMp-13a and CHMp-5b cells were suspended in serum-free RPMI-1640 medium and seeded into the upper chamber at a density of 5 × 10³ cells/well. HE extract (0-600 µg/mL) was added to the upper chamber, whereas 0.1% DMSO served as the negative control. The lower chamber contained RPMI-1640 medium supplemented with 10% FBS as a chemoattractant. After 24 h of incubation, nonmigrating cells were removed from the upper chamber, whereas migrated cells attached to the lower membrane surface were fixed with 100% MeOH and stained with Wright-Giemsa stain. Migrated cells were quantified by counting five random microscopic fields per insert, and the average values from three independent experiments were calculated.

### Cell apoptosis assay

Apoptosis induction by HE extract was quantified using the Annexin V-FITC apoptosis detection assay (BD Biosciences, San Jose, CA, USA) according to the manufacturer’s instructions. Briefly, 2 × 10^4^ CMC cells/well were seeded into 12-well plates and treated with HE extract at concentrations ranging from 0 to 600 µg/mL for 48 h. Cells treated with 0.1% DMSO served as the negative control, whereas cells treated with DOX (2 µg/mL) served as the positive control.

Following treatment, cells were detached and centrifuged at 400 × *g* for 5 min. Cell pellets were resuspended in 100 µL of binding buffer followed by the addition of 5 µL each of FITC Annexin V and propidium iodide (PI). Samples were analyzed using a CytoFLEX flow cytometer (Beckman Coulter, Miami, FL, USA), with 20,000 events collected per sample within 1 h of preparation. Quadrant gating strategies were established using unstained and single-stained controls (Supplementary Figure S2). To minimize spectral overlap between Annexin V-FITC and PI fluorescence signals, compensation matrixes were calculated and applied before data acquisition.

### RNA isolation, cdna synthesis, and qrt-PCR analysis

Total RNA was isolated from CMC cells treated with HE extract (0-600 µg/mL) using the GeneJET RNA Purification Kit (Thermo Fisher Scientific, Waltham, MA, USA) according to the manufacturer’s instructions. Complementary DNA (cDNA) synthesis was subsequently performed using the SuperScript III First-Strand Synthesis System (Invitrogen, Carlsbad, CA, USA) following previously described procedures [32, 33]. Thermal cycling was carried out using a G-Storm GS482 thermal cycler (Gene Technologies, Somerset, UK) under the following conditions: 65°C for 5 min, 50°C for 50 min, and 85°C for 5 min.

Quantitative real-time polymerase chain reaction (qRT-PCR) analysis was performed using a CFX96 Touch Real-Time PCR Detection System (Bio-Rad, Hercules, CA, USA) together with iTaq Universal SYBR Green Supermix (Bio-Rad) according to previously established protocols [33]. The relative mRNA expression levels of *BAX*, *BCL-2*, *SLUG*, *E-cadherin*, and *N-cadherin* were normalized against glyceraldehyde 3-phosphate dehydrogenase (*GAPDH)* using the 2^−ΔΔCq^ method. Amplification specificity was verified through dissociation curve analysis ranging from 65°C to 95°C. The primer sequences used in this study are presented in Table 1.

**Table 1:** Sequences of primers used for quantitative real-time polymerase chain reaction (qRT-PCR).

Gene	Primer sequence (5′-3′)	Amplicon size (bp)	Accession number	Reference
*BAX*	F: GGTTGTTGCCCTCCTCTACT R: GTAAGCACTCCAGCCACAAA	219	AB080230	[34]
*BCL-2*	F: TGGATGACTGAGTACCTGAA R: GGCCTACTGACTTCACTTAT	206	AB116145	[34]
*SLUG*	F: GGCAAGGCGTTTTCCAGACCCT R: GGGCAAGAAAAAGGCTTCTCCCCAG	77	NM_001097981.1	[35]
*E-cadherin*	F: TCCTGGGCAGGGTGAGTT R: GAGGCCGCTTGACTGTAATC	114	NM_001287125.2	[36]
*N-cadherin*	F: AGCACCCTCCTCAGTCAACG R: TGTCAACATGGTCCCAGCA	128	NM_001287156.2	[36]
*GAPDH*	F: CCCACTCTTCCACCTTCGAC R: AGCCAAATTCATTGTCATACCAGG	90	NM_001003142.2	[35]

### Western blot analysis

To evaluate the effects of HE extract on protein expression, CMC cells were treated with HE extract at concentrations ranging from 0 to 600 µg/mL for 48 h. Total protein was isolated using ice-cold radioimmunoprecipitation assay (RIPA) buffer (Cell Signaling Technology, Danvers, MA, USA). Cell lysates were sonicated and centrifuged at 14,000 × *g* for 10 min at 4°C to remove cellular debris. The supernatants were collected, and protein concentrations were quantified using a BCA Protein Assay Kit (Thermo Fisher Scientific, Grand Island, NY, USA).

Equal amounts of protein (20 µg) were separated by sodium dodecyl sulfate-polyacrylamide gel electrophoresis (SDS-PAGE) and subsequently transferred onto polyvinylidene difluoride (PVDF) membranes. Membranes were blocked and incubated overnight at 4°C with the following primary antibodies: anti-BAX (#AF0120; 1:2000), anti-BCL-2 (#AF6139; 1:5000), and anti-GAPDH (#AF7021; 1:5000) (Affinity Biosciences, Cincinnati, OH, USA), together with anti-E-cadherin (#14472; 1:2000), anti-N-cadherin (#13116; 1:2000), and anti-SLUG (#95858; 1:2000) (Cell Signaling Technology, Danvers, MA, USA).

After washing with tris-buffered saline containing 0.1% Tween-20, membranes were incubated with horseradish peroxidase (HRP)-conjugated anti-rabbit secondary antibody (#S0001; 1:5000) for 1 h at room temperatura (25°C). Protein bands were visualized using SuperSignal West Pico PLUS Chemiluminescent Substrate (Thermo Fisher Scientific) and detected using a ChemiDoc MP Imaging System (Bio-Rad) with a fixed exposure time of 1 min. Densitometric analysis was performed using Image Lab software (Bio-Rad). Protein expression levels were normalized to GAPDH as the internal loading control and expressed as percentages relative to the untreated control group.

### Phytochemical screening by LC/Q-TOF/MS

Phytochemical profiling of HE extract was performed using an LC/Q-TOF/MS system (6546 LC/Q-TOF; Agilent Technologies, Santa Clara, CA, USA) equipped with a Poroshell 120 EC-C18 column (2.7 µm, 3.0 × 150 mm). The analysis was conducted to identify potential bioactive compounds based on molecular mass and chromatographic retention characteristics.

The injection volume was set at 10 µL, with a flow rate of 0.7 mL/min. The column temperature was maintained at 40°C, whereas the autosampler tray temperature was maintained at 10°C throughout the analysis. Gradient elution was performed using a mobile phase consisting of 2 mM ammonium acetate in deuterium-depleted water (mobile phase A) and 100% MeOH (mobile phase B). The gradient program began with 2% mobile phase B for 1 min, gradually increased to 100% within 30 min, and was maintained for 7 min. The system was then returned to initial conditions for 1 min followed by a 3-min equilibration period, resulting in a total run time of 42 min.

Mass spectrometric analysis was conducted in both positive and negative ionization modes. Full-scan mass spectra were collected over an m/z range of 80-1000 at a scan rate of one scan/s.

### Statistical analysis

All experimental data are presented as mean ± standard deviation (SD). Statistical analyses for MTT assays were based on three independent experiments, with each treatment performed in quadruplicate. Wound healing assay data were obtained from four independent replicates. Quantitative data generated from Transwell migration assays, apoptosis assays, qRT-PCR analysis, and western blot analysis were derived from three independent experiments.

Data normality was assessed using the Shapiro-Wilk test. Comparisons between HE-treated groups and the control group (0.1% DMSO) were performed using one-way analysis of variance (ANOVA) followed by Dunnett’s multiple comparison test. Differences in IC_50_ values among cell lines and treatment durations were analyzed using the Extra sum-of-squares F-test. All statistical analyses were performed using GraphPad Prism version 8.0 (GraphPad Software Inc., La Jolla, CA, USA). Statistical significance was considered at p < 0.05.

## RESULTS

### Cytotoxic effects of HE extract

**Figure 1:**
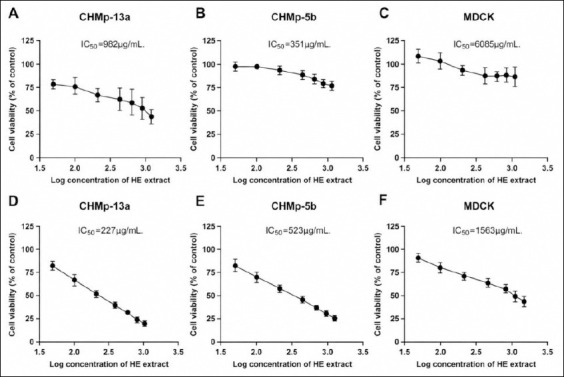
Dose-response curves of CHMp-13a, CHMp-5b, and MDCK cell proliferation following HE extract treatment. Cells were treated with varying concentrations (0-1000 µg/mL) of HE extract for 24 h (A-C) and 48 h (D-F). Data are presented as mean ± SD of three independent experiments.

The cytotoxic effects of HE extract against CMC cell lines and normal MDCK cells were evaluated using the MTT assay. Cells were treated with eight concentrations of HE extract (0-1000 µg/mL) for 24 and 48 h. The dose-response curves of CHMp-13a, CHMp-5b, and MDCK cells following treatment with HE extract are presented as percentages of cell viability in Figure 1. Detailed cell viability data and corresponding statistical analyses for all concentrations and time points are provided in Supplementary Tables S1.

Following both 24 and 48 h of treatment, HE extract demonstrated significant differences in IC_50_ values among the tested cell lines (p < 0.0001, Extra sum-of-squares F-test). At both time points, HE extract exhibited greater cytotoxic potency against CHMp-13a cells, with IC_50_ values of 982 and 227 µg/mL at 24 and 48 h, respectively, compared with CHMp-5b cells, which showed IC_50_ values of 3510 and 523 µg/mL, respectively. In contrast, minimal cytotoxicity was observed in normal MDCK cells, with IC_50_ values of 6085 and 1563 µg/mL at 24 and 48 h, respectively (Table 2).

**Table 2:** IC_50_ and SI values of HE extract against CHMp-13a, CHMp-5b, and MDCK cells following 24 and 48 h of treatment. Statistical significance was determined using the Extra sum-of-squares F-test.

Parameter	CHMp-13a		CHMp-5b		MDCK	
		
	24 h	48 h	24 h	48 h	24 h	48 h
IC_50_ values (µg/mL)	982	227	3510	523	6085	1563
95% confidence interval (95% CI)	774.9-1356	214.4-239.6	2750-4770	488.9-561	3314-16512	1329-1910
Selectivity index	6.19	6.92	1.73	2.98	-	-
p-value	< 0.0001	< 0.0001	< 0.0001	< 0.0001	-	-

The SI analysis indicated that an SI value >2 represents selective cytotoxicity toward cancer cells, whereas an SI value <1 indicates greater toxicity toward normal cells. Based on the calculated SI values, HE extract demonstrated selective inhibitory activity against both CMC cell lines. CHMp-13a cells exhibited high selectivity, with SI values of 6.19 and 6.92 at 24 and 48 h, respectively. In comparison, CHMp-5b cells exhibited lower selectivity, with SI values of 1.73 at 24 h and 2.98 at 48 h. These findings indicate that HE extract possesses stronger and more selective antiproliferative activity against CHMp-13a cells than against CHMp-5b cells.

### Effect of HE extract on the morphology of CMC cell lines

**Figure 2:**
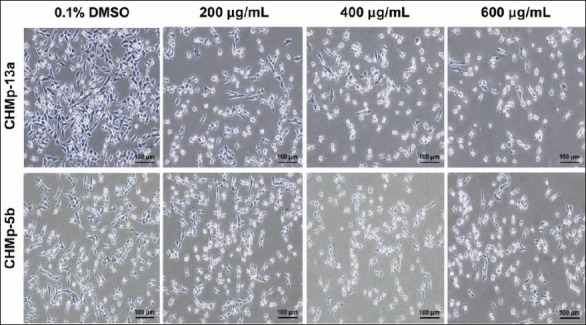
Morphological alterations in CHMp-13a and CHMp-5b cells following treatment with HE extract for 48 h. Concentration-dependent morphological changes were observed following HE extract treatment, including reduced cell population, cytoplasmic vacuolization, cell shrinkage, rounding, and detachment. Images were obtained using phase-contrast inverted microscopy at 10× magnification (scale bar = 100 µm).

Morphological alterations in CMC cells following treatment with HE extract were evaluated using phase-contrast microscopy after 48 h of incubation. As shown in Figure 2, HE extract induced concentration-dependent morphological changes in both CHMp-13a and CHMp-5b cells compared with untreated control cells. Microscopic examination demonstrated progressive reductions in cell density together with marked contraction of cell volume as the concentration of HE extract increased.

At the highest tested concentration (600 µg/mL), both CMC cell lines exhibited prominent cytoplasmic vacuolization, cell shrinkage, cellular rounding, detachment from the culture surface, and floating cells within the culture medium, all of which are characteristic morphological features associated with apoptosis. In contrast, untreated control cells maintained normal cellular morphology and adherence. These observations suggest that HE extract induces marked cytotoxic and apoptotic morphological alterations in CMC cells in a concentration-dependent manner.

### HE extract inhibits CMC cell migration

Cancer metastasis is a multistep process involving migration and invasion of tumor cells into surrounding tissues and distant organs. To investigate the antimigratory effects of HE extract, both scratch-wound healing and Transwell migration assays were performed.

**Figure 3:**
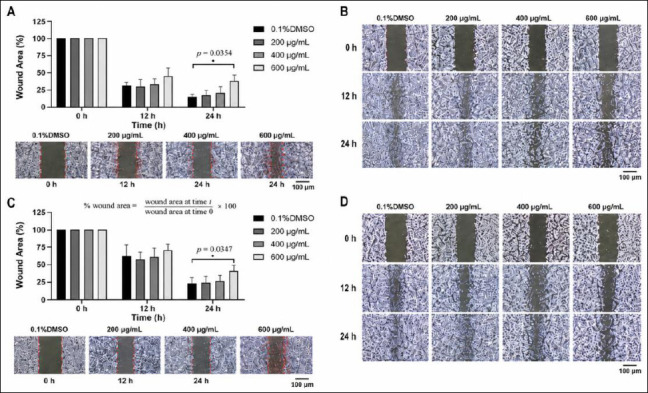
Evaluation of CHMp-13a and CHMp-5b cell migration using the wound healing assay following HE extract treatment. (A) Quantitative analysis of wound area in CHMp-13a cells and (C) CHMp-5b cells at 0, 12, and 24 h following treatment with 0-600 µg/mL HE extract. Data are expressed as mean percentage wound area ± SD of four independent replicates. Statistical analysis was performed using one-way ANOVA followed by Dunnett’s multiple comparison test (*p < 0.05 vs. control). (B) Representative wound healing images of CHMp-13a cells and (D) CHMp-5b cells captured at 4× magnification (scale bar = 100 µm).

The scratch-wound healing assay demonstrated that HE extract significantly inhibited wound closure in both CHMp-13a and CHMp-5b cells following 24 h of treatment (Figure 3). Specifically, treatment with 600 µg/mL HE extract significantly delayed scratch healing in CHMp-13a cells (p = 0.0354) and CHMp-5b cells (p = 0.0347) compared with the untreated control group. Detailed wound area measurements and statistical analyses are provided in Supplementary Tables S2.

**Figure 4:**
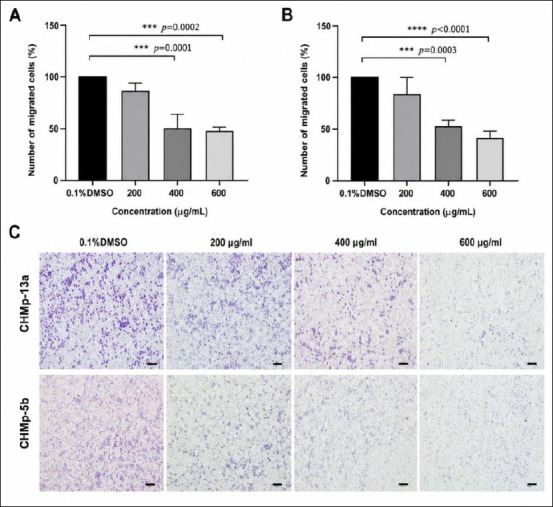
Evaluation of CHMp-13a and CHMp-5b cell migration using the Transwell assay following HE extract treatment. Cells were treated with HE extract (0, 200, 400, and 600 µg/mL) for 24 h. (A, B) Percentages of migrated cells relative to the untreated control are presented as mean ± SD of three independent replicates. Statistical analysis was performed using one-way ANOVA followed by Dunnett’s multiple comparison test (***p < 0.001 and ****p < 0.0001 vs. control). (C) Representative images of migrated cells captured at 4× magnification (scale bar = 100 µm).

Similarly, the Transwell migration assay demonstrated a significant concentration-dependent reduction in the migratory capacity of both CMC cell lines following HE extract treatment (Figure 4). In CHMp-13a cells, treatment with 400 and 600 µg/mL HE extract significantly reduced the percentage of migrated cells to 50.65% (p = 0.0002) and 48.46% (p = 0.0001), respectively, relative to the untreated control group (100%). Likewise, in CHMp-5b cells, treatment with 400 and 600 µg/mL HE extract significantly reduced cell migration to 53.45% (p = 0.0003) and 41.42% (p < 0.0001), respectively. Detailed migration cell counts and statistical analyses are provided in Supplementary Tables S3. These findings indicate that HE extract effectively suppresses the migratory behavior of CMC cells in a concentration-dependent manner.

### HE extract induces apoptosis in CMC cells

To determine whether HE extract induces apoptosis in CMC cells, Annexin V-FITC/PI double staining followed by flow cytometric analysis was performed. Cell populations were classified according to staining characteristics as viable cells, necrotic cells, early apoptotic cells, and late apoptotic cells.

**Figure 5:**
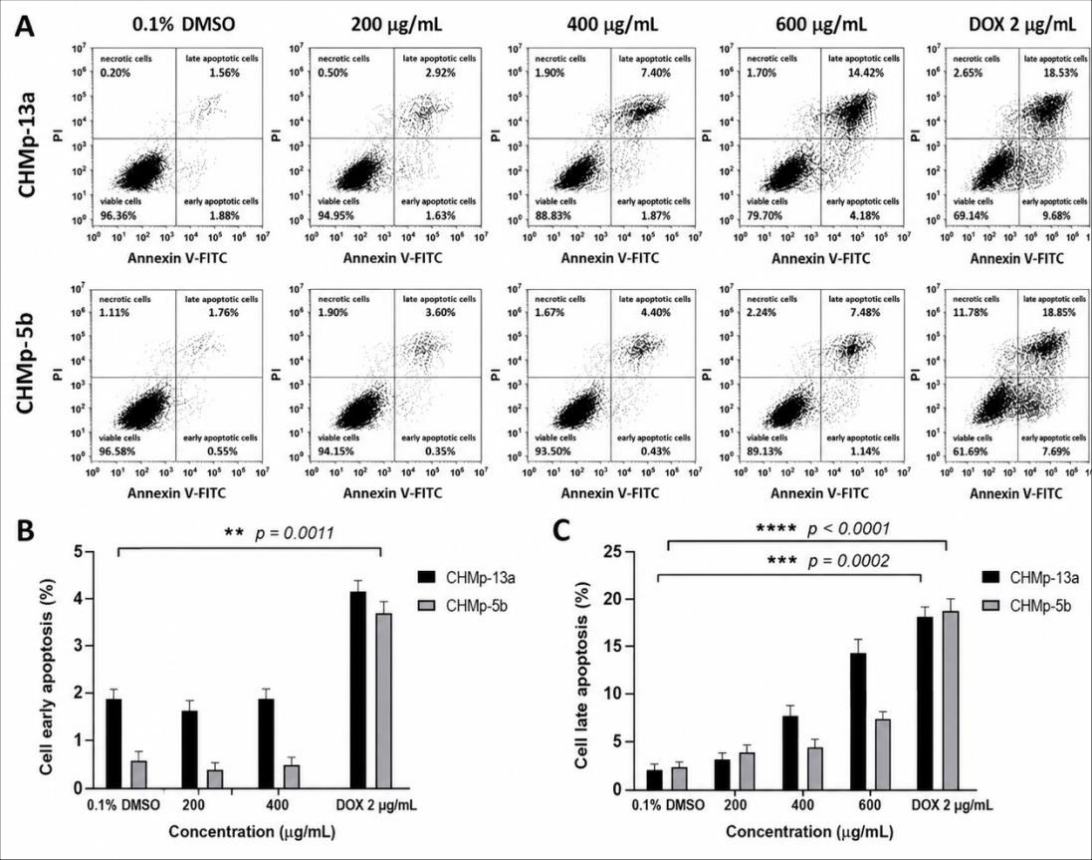
Flow cytometric quantification of apoptotic CHMp-13a and CHMp-5b cells following HE extract treatment. (A) Representative flow cytometry scatter plots showing Annexin V-FITC and PI staining after 48 h of treatment. (B) Percentages of early apoptotic cells (Annexin V+/PI−) and (C) late apoptotic cells (Annexin V+/PI+) relative to the total cell population. Data are presented as mean ± SD of three independent replicates. Statistical significance was determined relative to the untreated control group (**p < 0.01, ***p < 0.001, and ****p < 0.0001). DOX (2 µg/mL) and 0.1% DMSO served as positive and negative controls, respectively.

Treatment with HE extract (0-600 µg/mL) for 48 h induced a significant concentration-dependent increase in apoptotic cell populations in both CHMp-13a and CHMp-5b cells (Figure 5A). In CHMp-13a cells, treatment with 600 µg/mL HE extract significantly increased the percentages of early apoptotic and late apoptotic cells from 1.49% and 1.72% in untreated controls to 3.65% (p = 0.0011) and 17.97% (p = 0.0002), respectively (Figure 5B and 5C). Similarly, treatment of CHMp-5b cells with 600 µg/mL HE extract significantly increased late apoptotic cells from 2.00% in the untreated control group to 7.94% (p < 0.0001) (Figure 5C). Detailed apoptotic cell data and corresponding statistical analyses are provided in Supplementary Tables S4.

These findings demonstrate that HE extract effectively induces apoptosis in both CMC cell lines in a concentration-dependent manner.

### Relative mrna expression levels in CMC cells

**Figure 6:**
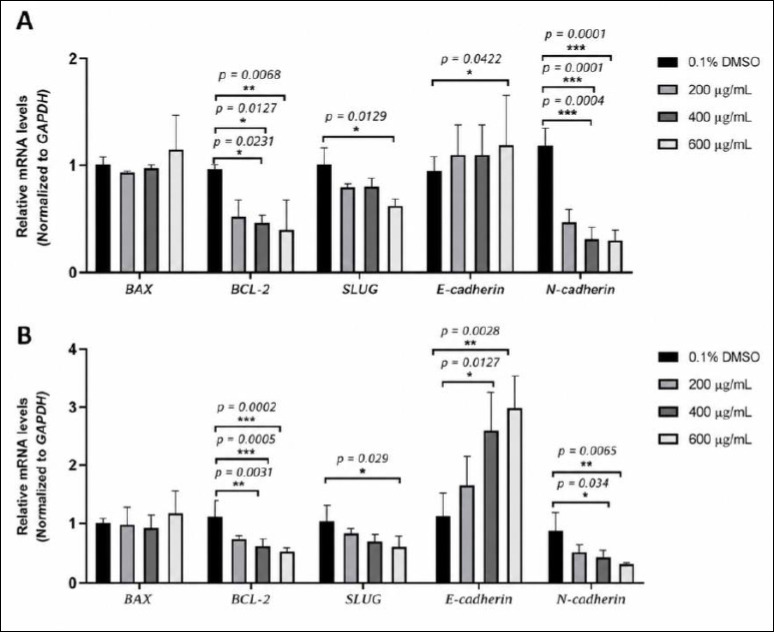
Relative mRNA expression levels in HE-treated CHMp-13a and CHMp-5b cells. Effects of HE extract on the expression of *BAX, BCL-2, SLUG, E-cadherin*, and *N-cadherin* in CHMp-13a (A) and CHMp-5b (B) cells following treatment with 0, 200, 400, and 600 µg/mL HE extract for 48 h. Relative mRNA expression levels were normalized to *GAPDH* expression. Data are presented as mean ± SD of three independent replicates. Statistical analyses were performed using one-way ANOVA followed by Dunnett’s multiple comparison test against the control group (0.1% DMSO). Statistical significance was considered at *p <0.05, **p < 0.01, and ***p < 0.001.

To elucidate the molecular mechanisms underlying HE extract-induced apoptosis and EMT modulation, the relative mRNA expression levels of apoptosis- and EMT-associated markers were quantified in both CMC cell lines using qRT-PCR analysis (Figures 6A and 6B).

In CHMp-13a cells, HE extract significantly reduced *BCL-2* mRNA expression at concentrations of 200 µg/mL (p = 0.0231), 400 µg/mL (p = 0.0127), and 600 µg/mL (p = 0.0068). Similarly, CHMp-5b cells exhibited significant downregulation of *BCL-2* expression following treatment with 200 µg/mL (p = 0.0031), 400 µg/mL (p = 0.0005), and 600 µg/mL (p = 0.0002) HE extract. In contrast, *BAX* mRNA expression demonstrated an increasing trend in response to escalating concentrations of HE extract in both CMC cell lines. These findings suggest that HE extract may promote apoptosis through modulation of apoptosis-regulating genes by shifting the *BAX/BCL-2* balance toward a proapoptotic state.

EMT is widely recognized as a critical biological process associated with enhanced invasiveness and metastatic dissemination of cancer cells [37]. A hallmark feature of EMT is the “cadherin switch,” characterized by reduced E-cadherin expression and increased N-cadherin expression, resulting in loss of intercellular adhesion and enhanced cellular motility [38]. To further investigate the inhibitory effects of HE extract on EMT, the expression levels of *E-cadherin, N-cadherin*, and *SLUG* were evaluated in both CMC cell lines.

Following treatment with HE extract, a marked shift in EMT-related gene expression profiles was observed. HE extract significantly increased *E-cadherin* expression in a concentration-dependent manner, indicating restoration of epithelial characteristics. In CHMp-13a cells, significant upregulation of *E-cadherin* expression was observed at 600 µg/mL (p = 0.0422). Similarly, in CHMp-5b cells, *E-cadherin* expression significantly increased at 400 µg/mL (p = 0.0127) and 600 µg/mL (p = 0.0028).

Conversely, expression of the mesenchymal markers *N-cadherin* and *SLUG* was significantly attenuated following HE extract treatment. In CHMp-13a cells, *N-cadherin* expression was significantly downregulated at 200 µg/mL (p = 0.0004), 400 µg/mL (p = 0.0001), and 600 µg/mL (p = 0.0001), whereas *SLUG* expression was significantly reduced at 600 µg/mL (p = 0.0129). In CHMp-5b cells, *N-cadherin* expression was significantly reduced at 400 µg/mL (p = 0.0340) and 600 µg/mL (p = 0.0065), whereas *SLUG* expression was significantly decreased at 600 µg/mL (p = 0.0290). Detailed mRNA expression data and statistical analyses are presented in Supplementary Tables S5.

Collectively, these findings suggest that HE extract may interfere with EMT progression in CMC cells by enhancing epithelial marker expression while suppressing mesenchymal-associated markers. These molecular alterations indicate the potential antimetastatic activity of HE extract in CMC cells. Further studies are warranted to elucidate the precise signaling pathways underlying the apoptosis-inducing and EMT-suppressive effects of HE extract.

### Protein expression by western blot analysis

To further evaluate the molecular effects of HE extract on apoptosis- and EMT-related pathways, western blot analysis was performed to determine the protein expression levels of BAX, BCL-2, E-cadherin, N-cadherin, and SLUG in both CMC cell lines following 48 h of treatment with HE extract.

**Figure 7:**
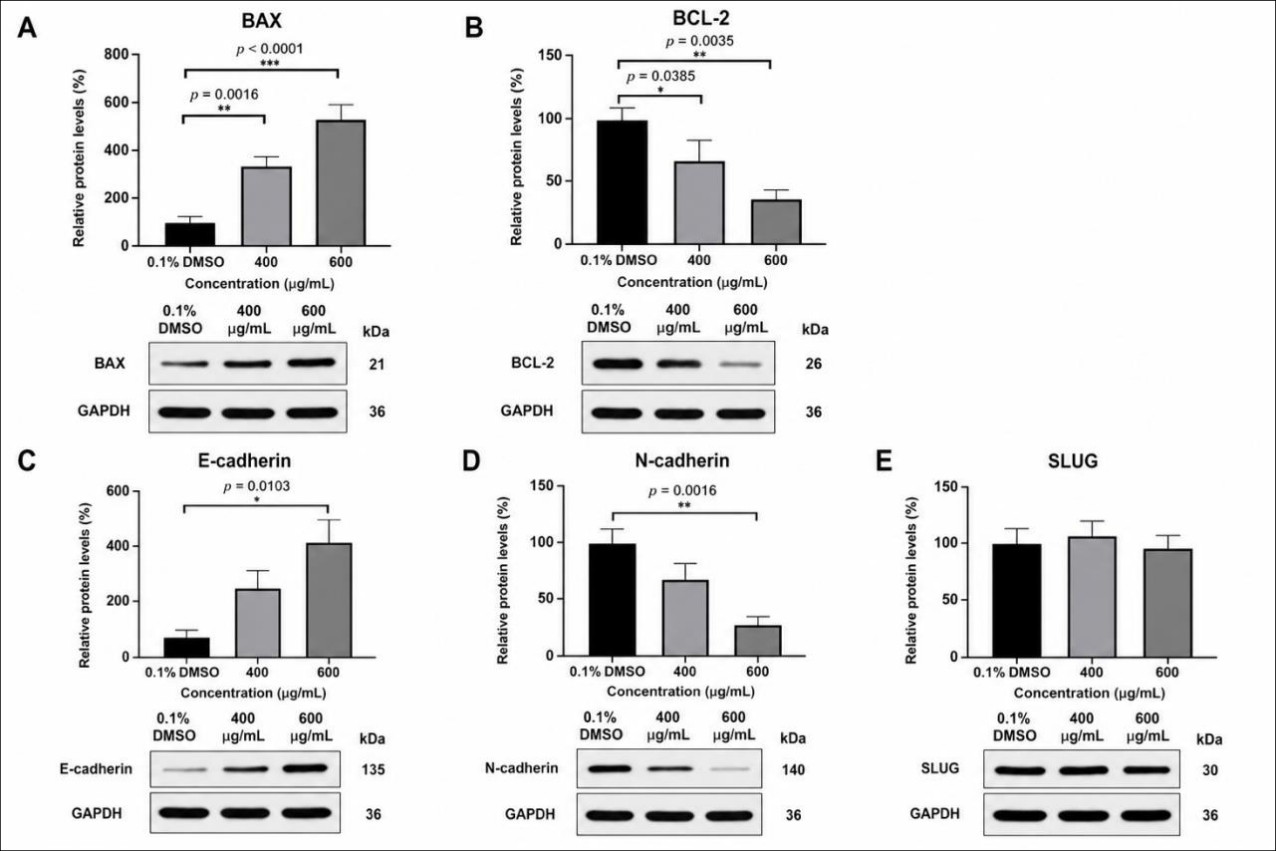
Western blot analysis of BAX, BCL-2, E-cadherin, N-cadherin, and SLUG proteins in CHMp-13a cells following HE extract treatment. (A-E) Cells were treated with 0, 400, and 600 µg/mL HE extract for 48 h. Protein expression levels were normalized to GAPDH expression. Data are presented as mean ± SD of three independent replicates. Statistical analyses were performed using one-way ANOVA followed by Dunnett’s multiple comparison test against the control group (0.1% DMSO). Statistical significance was considered at *p <0.05, **p < 0.01, ***p < 0.001, and ****p<0.0001.

**Figure 8:**
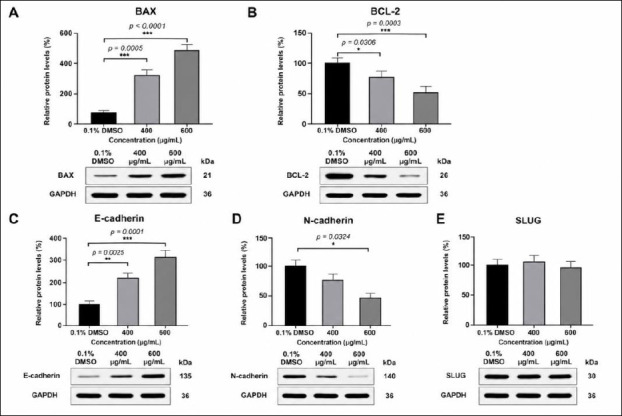
Western blot analysis of BAX, BCL-2, E-cadherin, N-cadherin, and SLUG proteins in CHMp-5b cells following HE extract treatment. (A-E) Cells were treated with 0, 400, and 600 µg/mL HE extract for 48 h. Protein expression levels were normalized to GAPDH expression. Data are presented as mean ± SD of three independent replicates. Statistical analyses were performed using one-way ANOVA followed by Dunnett’s multiple comparison test against the control group (0.1% DMSO). Statistical significance was considered at *p <0.05, **p < 0.01, ***p < 0.001, and ****p<0.0001.

Alterations in BCL-2 family proteins play essential roles in regulating apoptosis [39]. In CHMp-13a cells (Figures 7A and 7B), HE extract significantly increased BAX protein expression at 400 µg/mL (p = 0.0016) and 600 µg/mL (p < 0.0001), whereas BCL-2 protein expression was significantly decreased at the same concentrations (p = 0.0385 and p = 0.0035, respectively). Similarly, in CHMp-5b cells (Figures 8A and 8B), HE extract significantly elevated BAX protein expression at 400 µg/mL (p = 0.0005) and 600 µg/mL (p < 0.0001), accompanied by significant reductions in BCL-2 expression at 400 µg/mL (p = 0.0306) and 600 µg/mL (p = 0.0003). These findings support the involvement of HE extract in promoting apoptosis through modulation of intrinsic apoptotic pathways.

To further investigate the role of HE extract in suppressing metastatic potential, the expression of EMT-associated proteins was also examined. In CHMp-13a cells, E-cadherin expression was significantly increased at 600 µg/mL (p = 0.0103), whereas N-cadherin expression was significantly reduced at the same concentration (p = 0.0016) (Figures 7C and 7D). Similarly, in CHMp-5b cells, E-cadherin expression significantly increased at 400 µg/mL (p = 0.0025) and 600 µg/mL (p = 0.0001), whereas N-cadherin expression was significantly decreased at 600 µg/mL (p = 0.0324) (Figures 8C and 8D).

In contrast, no significant alterations in SLUG protein expression were detected in either cell line at any tested concentration (Figures 7E and 8E). Detailed protein expression data, statistical analyses, and uncropped western blot images are presented in Supplementary Tables S6 and Supplementary Figure S3. Overall, these findings suggest that HE extract modulates the metastatic phenotype of CMC cells through suppression of EMT-associated molecular alterations together with regulation of intrinsic apoptotic signaling pathways mediated by BCL-2 family proteins.

### Phytochemical screening analysis

Phytochemical characterization of the methanolic HE extract using LC/Q-TOF/MS in both positive and negative ionization modes revealed the presence of multiple bioactive compounds. A total of 17 phytochemical compounds with potential anticancer activity were identified based on retention time (Rt), peak intensity, and molecular mass analysis (Table 3 and Supplementary Figure S4).

**Table 3:** Identified phytochemical compounds in HE extract determined by liquid chromatography quadrupole time-of-flight mass spectrometry (LC/Q-TOF/MS).

No.	Identified compounds	Rt (min)	Formula	[M+H]+ (m/z)	[M+H]− (m/z)	MS/MS fragment ion (m/z)
1	Herierin IV	5.722	C8H10O4	170.0584	-	171.0657, 172.0688, 193.0475
2	Herniarin	12.950	C10H8O3	177.0546	-	177.0554, 194.0821, 199.0366
3	Gingerenone B	13.008	C22H26O6	386.1739	-	387.1802, 404.2068, 409.1622
4	Erinacerin G	16.542	C16H19NO5	305.1292	-	306.1336, 323.1601, 328.1155
5	Coriandrone E	18.515	C13H12O5	248.0689	-	249.0757, 266.1023, 271.0577
6	Calomelanol J	20.253	C24H18O5	386.1139	-	387.1227, 404.1492, 409.1046
7	4-Gingerol	20.600	C15H22O4	266.1523	-	267.1591, 284.1856, 289.1410
8	Uncarine F	21.328	C21H24N2O4	368.1741	-	369.1809, 386.2074, 391.1628
9	Garcinone E	22.920	C28H32O6	464.2217	-	465.2272, 466.2306, 482.2537, 483.2570
10	Erinacine A	25.888	C25H36O6	432.2532	-	433.2585, 434.2619, 450.2850, 451.2883
11	Hericenone B	27.661	C27H31NO4	433.2267	-	434.2326, 435.2359, 451.2591, 452.2624
12	8-Gingerol	28.033	C19H30O4	322.2146	-	323.2217, 340.2482, 345.2036
13	Hericerin	30.593	C27H33NO3	419.2468	-	420.2533, 421.2566, 438.2831
14	Hericenone F	31.087	C35H54O6	570.3954	-	571.3993, 572.4027, 588.4259, 589.4292
15	Sesaminol 2-O-triglucoside	31.989	C36H46O22	830.2511	-	831.2588, 848.2819, 849.2853
16	Hericene A	33.319	C35H56O5	-	556.4106	557.4201, 558.4235, 574.4466, 575.4500
17	Hericene B	34.587	C37H58O5	-	582.4294	583.4357, 584.4391, 600.4623, 601.4656

Among the identified compounds, hericenones and erinacines represented the major classes of monoterpenoid compounds. These compounds have previously been associated with diverse biological activities, including antioxidant, anticancer, neuroprotective, immunoregulatory, antibacterial, and hypoglycemic effects [40]. In addition, hericene and hericerin, which are classified as geranyl resorcinol meroterpenoids predominantly identified in *Hericium* species, were also detected and are believed to possess multiple beneficial pharmacological activities [41].

Several additional bioactive constituents, including phenolic compounds, polyphenols, pyranones, terpenoid alkaloids, xanthones, benzaldehydes, and oligosaccharides, were also identified in HE extract. The presence of these diverse phytochemicals suggests that HE extract possesses a complex phytochemical composition that may contribute synergistically to its observed anticancer activities. These findings further support the potential therapeutic application of HE extract as a source of bioactive compounds for future functional and mechanistic anticancer studies.

## DISCUSSION

### Therapeutic challenges in CMC

Mammary cancer is one of the most frequently diagnosed neoplastic diseases in women and female dogs and remains a major public health and veterinary concern worldwide. CMC is a multifactorial disease influenced by several genetic, hormonal, environmental, and nutritional factors that contribute to tumor initiation and progression [42]. As one of the most prevalent cancers in dogs, CMC markedly affects survival rate and quality of life, thereby creating substantial therapeutic challenges for veterinary clinicians. Surgical excision remains the principal treatment modality for localized mammary tumors and commonly involves removal of the tumor together with regional lymph node dissection to minimize metastatic spread. In advanced or metastatic cases, chemotherapy is often administered as adjuvant or palliative therapy following surgery to reduce recurrence and improve overall survival [43, 44]. Nevertheless, conventional chemotherapeutic regimens are frequently associated with adverse side effects and variable therapeutic outcomes. Consequently, increasing attention has been directed toward the identification of alternative therapeutic agents or adjuvant strategies capable of enhancing treatment efficacy while minimizing toxicity and improving patient quality of life.

### Anticancer potential of medicinal mushrooms and HE extract

Medicinal mushrooms possess diverse biological activities, including antimicrobial, anticancer, immuno-modulatory, antioxidant, anti-inflammatory, antidiabetic, and prebiotic properties [45, 46]. Importantly, these natural products are generally associated with low toxicity and minimal adverse effects, making them attractive candidates for novel therapeutic development [47, 48]. Numerous studies have demonstrated that mushroom-derived compounds such as phenolic acids, polyphenols, flavonoids, polysaccharides, and terpenoids modulate critical molecular pathways involved in cancer initiation, progression, apoptosis, metastasis, and oxidative stress regulation [49, 50]. For example, Li *et al*. [51] reported that HE ethanol extract exhibited cytotoxic activity against Huh-7, HepG2, HT-29, and NCI-87 cancer cell lines with IC_50_ values of 80, 2500, 1250, and 4500 µg/mL, respectively. Furthermore, HE extract demonstrated significant antitumor efficacy in four *in vivo* xenograft models without detectable toxicity in mice [52]. Despite these promising findings, studies investigating the anticancer effects of HE extract in canine cancers, particularly CMC, remain extremely limited. Previous mechanistic investigations in human cancer cells have shown that HE extract may induce apoptosis through modulation of proapoptotic and antiapoptotic proteins [53], activation of caspase pathways [54], reactive oxygen species (ROS) generation [55], inhibition of tumor metastasis [56], and suppression of angiogenesis [57]. However, these molecular effects have not previously been characterized in CMC models.

### Selective antiproliferative activity of HE extract

In the present study, HE extract demonstrated potent antiproliferative activity against both CHMp-13a and CHMp-5b CMC cell lines, with greater sensitivity observed in CHMp-13a cells. Following 48 h of treatment, the IC_50_ values were 227 µg/mL for CHMp-13a cells and 523 µg/mL for CHMp-5b cells. In contrast, normal MDCK cells exhibited substantially higher IC_50_ values (1563 µg/mL), indicating relatively low cytotoxicity toward noncancerous cells. Moreover, SI values of 6.92 for CHMp-13a cells and 2.98 for CHMp-5b cells further confirmed the selective cytotoxic activity of HE extract toward malignant cells while sparing normal cells at therapeutic concentrations. These findings highlight the potential application of HE extract as a selective therapeutic or adjuvant agent in canine mammary oncology.

The observed IC_50_ values obtained in this study are consistent with previous findings in human breast cancer models. Atay *et al*. [30] demonstrated that HE water extract inhibited MCF-7 breast cancer cell proliferation with an IC_50_ value of 250 µg/mL after 72 h of treatment. Although our findings in CMC cells (IC_50_ = 227-523 µg/mL) closely resemble these previously reported values, differences in extraction methodology and treatment duration may influence the biological activity of the extracts. In the previous study, dose-dependent inhibition was most prominent after 72 h, whereas the methanolic HE extract used in our study exerted significant antiproliferative effects within 48 h. These observations suggest that both extraction solvent and exposure duration may substantially influence the anticancer efficacy of HE extract. Several studies have proposed that terpenoids and polyphenols present in mushroom extracts contribute significantly to antioxidant and antiproliferative activities [58-60]. In particular, terpenoids identified in HE extracts exhibit cytotoxic effects against multiple human cancer cell lines [61]. Therefore, the antiproliferative activity observed in this study may be associated with the presence of bioactive compounds such as erinacines, hericenones, hericene A, and hericerin identified during LC/Q-TOF/MS analysis.

### Inhibition of CMC cell migration and EMT modulation

Metastasis is a critical step in cancer progression and involves migration and invasion of tumor cells from the primary site to distant organs [62]. Therefore, inhibition of tumor migration represents an important therapeutic target for limiting metastatic dissemination. In the present study, both scratch-wound healing and Transwell migration assays demonstrated that HE extract significantly suppressed migration of CHMp-13a and CHMp-5b cells in a concentration-dependent manner. Treatment with 400 µg/mL HE extract reduced migration by approximately 50%, whereas 600 µg/mL markedly delayed wound closure and significantly inhibited cell migration in both CMC cell lines. These findings indicate that HE extract possesses substantial antimetastatic activity against CMC cells.

EMT is widely recognized as a major biological mechanism contributing to cancer metastasis and tumor aggressiveness [63]. This process is characterized by downregulation of epithelial markers such as E-cadherin and upregulation of mesenchymal markers including N-cadherin and SLUG, leading to loss of cell-cell adhesion and enhanced cellular motility [64]. In this study, HE extract significantly increased E-cadherin expression while simultaneously suppressing N-cadherin expression at both mRNA and protein levels in CHMp-13a and CHMp-5b cells. Furthermore, significant reductions in *SLUG* mRNA expression were observed following treatment with 600 µg/mL HE extract. However, no significant changes in SLUG protein expression were detected by western blot analysis. These findings suggest that SLUG may not represent the primary mediator responsible for EMT suppression in the present experimental conditions. Nevertheless, posttranslational modifications or temporal variations in SLUG regulation cannot be excluded [65, 66]. Since EMT-associated transcription factors may exhibit dynamic temporal expression patterns, the 48 h treatment period used in this study may not correspond to the peak period of SLUG degradation. Therefore, future investigations employing broader temporal analyses are required to clarify the precise role of SLUG during HE-mediated EMT suppression. Despite stable SLUG protein levels, the observed increase in E-cadherin together with reduction in N-cadherin strongly indicates that HE extract interferes with EMT progression, potentially through alternative regulatory pathways independent of classical SLUG-mediated signaling. Similar findings have previously been reported in CT-26 colon cancer-transplanted mice, where HE extract significantly reduced lung metastasis through suppression of matrix metalloproteinases and EMT-associated pathways [56]. Although these findings support the antimetastatic potential of HE extract, the absence of pathway-specific inhibitor studies remains a limitation of the present work. Future studies using EMT-related signaling inhibitors are necessary to identify the precise upstream molecular pathways involved in HE-mediated EMT regulation.

### Apoptosis induction through BAX/BCL-2 regulation

Induction of apoptosis is one of the principal strategies in cancer therapy for preventing tumor progression, recurrence, and metastasis [67]. Morphological characteristics of apoptosis include cell rounding, shrinkage, detachment, cytoplasmic vacuolization, and loss of intercellular adhesion [68, 69]. In the present study, HE extract induced distinct apoptotic morphological alterations in both CMC cell lines, including cytoplasmic vacuolization, cell shrinkage, rounding, and reduced cell-cell adhesion. These findings were further supported by Annexin V-FITC/PI flow cytometric analysis, which demonstrated significant increases in apoptotic cell populations following treatment with HE extract. Treatment with 600 µg/mL HE extract for 48 h significantly increased late apoptotic cell populations in both CHMp-13a and CHMp-5b cells compared with the 0.1% DMSO control group. These observations indicate that HE extract effectively induces apoptosis in CMC cells.

Mitochondrial-mediated apoptosis represents the predominant form of programmed cell death in mammalian cells and is tightly regulated by members of the BCL-2 family proteins [70]. Proapoptotic proteins include BAX, Bcl-2 homologous antagonist/killer, Bcl-2-related ovarian killer, and Bcl-2-associated agonist of cell death, whereas antiapoptotic proteins include BCL-2, B-cell lymphoma-w, and B-cell lymphoma-extra-large [71]. In the present study, HE extract significantly increased BAX expression while simultaneously decreasing BCL-2 expression at both mRNA and protein levels in both CMC cell lines. These findings suggest that HE extract induces apoptosis primarily through modulation of the mitochondrial apoptotic pathway by shifting the BAX/BCL-2 ratio toward a proapoptotic state. Similar observations have previously been reported in human U937 leukemia cells, where HE extract activated mitochondria-mediated apoptotic signaling and increased the ratio of proapoptotic to antiapoptotic proteins [49]. Nevertheless, additional mechanistic studies involving caspase activation assays and ROS quantification are required to further distinguish the involvement of intrinsic and extrinsic apoptotic pathways in HE-treated CMC cells.

### Phytochemical contribution to anticancer activity

The anticancer activity of HE extract observed in this study may be attributed to its diverse phytochemical composition, including terpenoids, phenolic compounds, flavonoids, alkaloids, and polysaccharides, which are known to exert antioxidant, cytotoxic, immunomodulatory, and antimetastatic effects [72]. In particular, erinacines and hericenones are considered the major therapeutically active compounds in HE mushrooms and have previously been shown to regulate mitochondrial apoptosis through modulation of the BAX/BCL-2 ratio in colorectal cancer cells. Erinacine A has been reported to activate both intrinsic and extrinsic apoptotic pathways in DLD-1 and HCT-116 colorectal cancer cells [73]. Furthermore, Ruan *et al*. [74] demonstrated that hericenone exhibits cytotoxic activity against HCT-116 colorectal carcinoma and HepG2 liver cancer cells. Additional studies have also shown that HE extract enhances sensitivity of HepG2 and SGC7901 cells to DOX-induced apoptosis [75, 76]. Moreover, *in vivo* investigations demonstrated that HE extract reduced tumor size and metastasis in mice inoculated with 26-M3.1 colon cancer cells [77]. Collectively, these findings support the hypothesis that the biological activities of HE extract result from synergistic interactions among multiple bioactive constituents acting through multitargeted molecular mechanisms.

### Strengths, limitations, and future perspectives

Previous toxicological investigations further support the safety profile of HE extracts. Studies evaluating aqueous and methanolic HE extracts in isolated mouse hepatocytes reported minimal cytotoxicity [78]. Furthermore, long-term toxicity studies demonstrated that HE powder produced no adverse effects in rats even at doses of 2000 mg/kg administered over 90 days [79]. These findings suggest that HE extract may represent a promising candidate for development as a potential adjuvant therapeutic agent for CMC management.

One of the major strengths of the present study is the comprehensive evaluation of HE extract using multiple biological approaches, including antiproliferative assays, migration assays, apoptosis analysis, EMT-associated molecular evaluation, western blot analysis, and phytochemical profiling. Additionally, the use of two distinct CMC cell lines with different invasive phenotypes provides broader insight into the therapeutic potential of HE extract in canine mammary oncology. The integration of LC/Q-TOF/MS phytochemical characterization further strengthens the translational relevance of the study by identifying multiple bioactive compounds potentially associated with the observed anticancer activities.

However, several limitations should also be acknowledged. First, the present study was conducted entirely under *in vitro* conditions and therefore does not fully replicate the complex tumor microenvironment or systemic physiological interactions present in living animals. Second, *in vivo* pharmacokinetic, toxicity, and efficacy data are currently lacking. Third, possible batch-to-batch variation in phytochemical composition may influence biological reproducibility. Additionally, the absence of pathway-specific inhibitor studies limits mechanistic interpretation of EMT and apoptosis regulation.

Therefore, future investigations should focus on standardization of HE extract through identification and quantification of key bioactive compounds, evaluation of ROS generation and caspase activation, assessment of potential synergistic interactions with conventional chemotherapeutic agents such as DOX, and *in vivo* validation of safety and therapeutic efficacy in animal models. Furthermore, advanced formulation strategies such as nanoencapsulation may improve bioavailability and enhance clinical translatability of HE extract for veterinary oncology applications.

## CONCLUSION

This study demonstrated that HE extract possesses significant *in vitro* anticancer activity against CMC cell lines, particularly CHMp-13a cells, through selective antiproliferative, antimigratory, and proapoptotic effects. HE extract exhibited greater cytotoxic selectivity toward malignant CMC cells than normal MDCK cells, as evidenced by favorable SI values. Morphological observations together with Annexin V-FITC/PI analysis confirmed that HE extract effectively induced apoptosis in both CMC cell lines. Furthermore, molecular analyses revealed that HE extract promoted apoptosis through upregulation of BAX and suppression of BCL-2 expression, suggesting involvement of the mitochondrial apoptotic pathway. In addition, HE extract significantly inhibited migration and partially suppressed EMT by increasing E-cadherin expression while decreasing N-cadherin expression in both CMC cell lines.

The phytochemical profiling by LC/Q-TOF/MS identified multiple bioactive compounds, including erinacines, hericenones, hericene A, and hericerin, which may collectively contribute to the observed anticancer activities. These findings highlight the therapeutic potential of HE extract as a promising natural source of bioactive compounds for CMC management. A major strength of this study was the integrated evaluation of antiproliferative activity, apoptosis induction, EMT modulation, protein expression, and phytochemical characterization using two CMC cell lines with distinct invasive phenotypes.

Nevertheless, this study also has several limitations. The experiments were conducted entirely under *in vitro* conditions and therefore do not fully represent the complexity of the tumor microenvironment and systemic interactions occurring *in vivo*. Moreover, the absence of pharmacokinetic, toxicological, ROS-related, and caspase activation analyses limits comprehensive mechanistic interpretation of the anticancer effects of HE extract. Variability in phytochemical composition among extract batches may also affect reproducibility.

Future studies should focus on *in vivo* validation of safety and therapeutic efficacy, identification and standardization of the major active compounds, investigation of ROS and caspase signaling pathways, and evaluation of potential synergistic interactions between HE extract and conventional chemotherapeutic agents such as DOX. Additionally, advanced delivery systems including nanoformulations may further improve the clinical applicability and bioavailability of HE extract.

Collectively, the findings of this study suggest that HE extract may serve as a promising candidate for development as an adjuvant therapeutic agent for canine mammary cancer by promoting apoptosis and suppressing EMT-associated migration in CMC cells.

## DATA AVAILABILITY

The supplementary data can be made available from the corresponding author upon request.

## AUTHORS’ CONTRIBUTIONS

UJ: Conceptualization, methodology, software, validation, formal analysis, investigation, data curation, writing – original draft preparation, writing – review and editing, and project administration. AS: Methodology, formal analysis, validation, data curation, and writing – original draft preparation. TJ: Supervision, methodology, and writing – original draft preparation. SK: Conceptualization, supervision, and visualization. SS: Methodology and writing – review and editing. WS: Methodology and writing – review and editing. KS: Supervision, visualization, and data curation. AK: Investigation and writing – original draft preparation. YBY: Methodology, formal analysis, investigation, data curation, and writing – original draft preparation. All authors have read and approved the final version of the manuscript.
